# *Plasmodium falciparum* Clinical Isolates Reveal Analogous Circulation of 3D7 and FC27 Allelic Variants and Multiplicity of Infection in Urban and Rural Settings: The Case of Adama and Its Surroundings, Oromia, Ethiopia

**DOI:** 10.1155/2022/5773593

**Published:** 2022-03-14

**Authors:** Temesgen File, Lemu Golassa, Hunduma Dinka

**Affiliations:** ^1^Department of Applied Biology, Adama Science and Technology University, P.O. Box 1888, Adama, Ethiopia; ^2^Aklilu Lemma Institute of Pathobiology, Addis Ababa University, P.O. Box 1176, Addis Ababa, Ethiopia

## Abstract

**Background:**

Despite significant progress achieved globally in reducing malaria burden, still it is one of the major public health and economic problems in Ethiopia. Investigation of the local genetic polymorphism of *P. falciparum*, the most virulent and predominant malaria parasite primarily targeted in malaria control and elimination program, is paramount to assess intensity of parasite transmission. Analysis of the block 3 region of the *msp-2* gene of *P. falciparum* provides strong molecular evidence to evaluate the real picture of malaria epidemiology to fine-tune the ongoing control and elimination programs in the region. Thus, this study was aimed at examining the status of such polymorphic gene and its implications in Adama and its surroundings.

**Methods:**

148 isolates from patients with uncomplicated falciparum malaria were collected in the study from September 2019 to August 2020. Tween® 20 and the Chelex method were employed for parasite DNA extraction. *msp-2* allelic families were genotyped by using nested polymerase chain reaction targeting its 3D7 and FC27 allelic variants followed by gel electrophoresis for fragment analysis.

**Results:**

Seventeen different polymorphic forms of msp-2 allelic fragments were detected in the study area. Moreover, 47 (31.8%) and 41(27.7%) were detected for 3D7 and FC27 allelic families, respectively. Furthermore, the multiclonal allele type accounted for 60 (40.5%). The mean MOI was 1.4, and the heterogeneity index (He) is 0.49 indicating nearly intermediate malaria transmission in the study area.

**Conclusions:**

The study revealed nearly intermediate genetic diversity and mean MOI of *P. falciparum* in the study area, demanding further scale up of the ongoing control and elimination efforts.

## 1. Introduction

World Health Organization (WHO) report shows that in 2019, the number of malaria cases was 229 million, out of which 409,000 deaths were recorded. More than 90% of all malaria cases and death were only from sub-Saharan Africa (SSA) [1]. In Ethiopia, malaria is responsible for a major public health and economic problem. In 2017/18 alone, the number of confirmed malaria cases reported was 1,206,892. Of this, 883,886 (69.2%) and 181,964 (30.8%) were *P. falciparum* and *P. vivax*, respectively [2]. Its distribution varies from place to place due to the variation in climatic factors, rainfall patterns, and altitude [3]. Malaria is one of the leading causes of morbidity and mortality in Ethiopia; it is prevalent in 75% of the country's land mass, where 68% of the population inhabits. *P. vivax* and *P. falciparum* are the two coendemic species in the country, sharing the overall burden of 30% and 70%, respectively [3, 4]. Similarly, it was reported that malaria is the third leading cause of outpatient department (OPD) visits (36%) in the East Shawa Zone of the Oromia Regional State [5], which includes the present study area. The five-year trend analysis of malaria cases in three districts (Adama, Lume/Modjo, and Boset/Olanciti) in Central Ethiopia showed microscopy slide positivity rate reduction from 16.3% in 2016 to 10.1% in 2020 [6].

In Ethiopia, even though the various intervention efforts made so far have significantly reduced the disease burden, this promoted the country to move forward and plan for malaria elimination strategies in selected areas [7]. However, malaria is remaining to be one of the major health problems in the country. The major hurdle that limited the national effort is the challenges related to effective implementation of major intervention strategies for malaria control like early diagnosis, prompt treatment, selective vector control, and environmental management together with resource intensive nature of the programs [5, 7]. In addition, studies also show that a number of factors like the emergence of insecticide resistance by the mosquito vectors, increased population density, global warming, poverty, the lack of effective vaccines, and the emergence and spread of drug-resistant strains could limit malaria control and elimination plan [8, 9]. Furthermore, the frequent emergence and spread of genetic diversity of the predominant malaria parasite *P. falciparum* are another challenge.

In a malaria endemic region, *P. falciparum* infection is characterized by having higher genetic diversity, which is implicated in its evolutionary fitness, and consequently having more survival advantage by overcoming the control efforts [10]. Genetic diversity is not only an indicator of the intensity of transmission [11, 12] but also an indicator of how effective control and intervention are. Likewise, high genetic diversity in an area with substantial control options implicates that the control program is not effective in reducing parasite population and vice versa. Therefore, understanding the genetic structure of a parasite population can complement the implementation of malaria control interventions.

The most commonly used technique for molecular characterization of such polymorphism in a malaria endemic area is the use of nested amplification of merozoite surface proteins (MSP) like the block 3 region of the *msp-2* gene [13, 14]. MSP-2 is a glycoprotein approximately 30 kDa encoded by the *msp-2* gene located on chromosome 2. It is composed of five blocks of which the central block (block 3) is the most polymorphic part [15, 16]. Thus, *msp-2* has been used extensively to describe the parasite populations as a discriminatory and informative marker for strain differentiation [8]. Due to the variable nonrepeat sequences as well as the varying sizes of the tandem repeats in the central region, the *msp-2* gene is dimorphic and existing in two main allelic families: FC27 and 3D7 [14, 17]. Therefore, molecular-based investigation of the diversity of *P. falciparum* and its multiplicity of infection (MOI) describes the level of malaria transmission and the effectiveness of ongoing malaria control and elimination strategy. For successful malaria elimination, information on the genetic profile of the parasite population in different geographical areas and factors that determine gene flow between locations is paramount. Even though genetic diversity of *P. falciparum* has been extensively studied in different parts of the world, there is limited information in Ethiopia. Therefore, the aim of this study was to examine genetic polymorphism of the block 3 region of the *msp-2* gene of *P. falciparum* and its MOI in Adama town and its surroundings, Oromia Regional State, Central Ethiopia. Attempt was made to analyze the spatial and temporal feature of such polymorphism in the study area and its comparison with different localities showing brunt of falciparum infection and endemicity.

## 2. Materials and Methods

### 2.1. Study Sites

**Figure 1:**
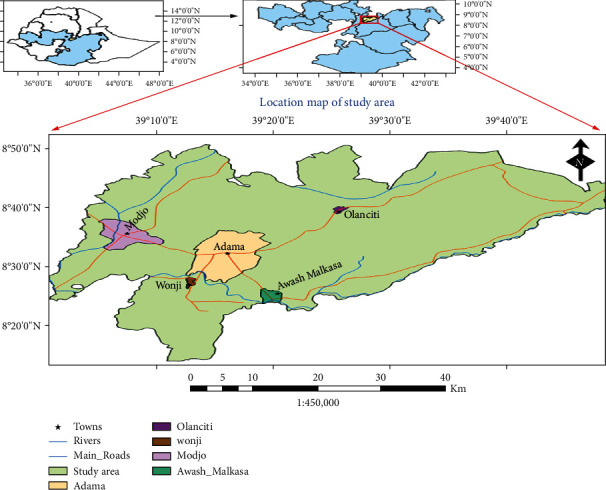
Map of the study area (figure developed by using Arc-GIS Desktop version 10.4).

The study samples were collected from three districts in Central Ethiopia (Adama, Modjo, and Olanciti), including Adama town administration from September 2019 to August 2020. Adama is the major town next to the main capital in Ethiopia, located at a distance of about 99 km southeast of Addis Ababa. Adama District includes Wonji located at 8 km south and Awash Melkasa situated at a distance of 15 km southeast of Adama. Other health facilities are located in neighboring districts like Modjo located at 16 km northwest and Olanciti situated at 23 km northeast of Adama. The population projection of the catchment study area estimated to reach 800,000 inhabitants. Adama and its neighboring districts are located in the rift valley area of Central Ethiopia (Figure 1), where malaria is endemic. Similar to the other part of the country where malaria is endemic, malaria transmission in the study area is seasonal based on the rainfall patterns that are heavy from mid-June to mid-September which accounts for major malaria transmission season from mid-September to December. And a shorter rainy period near April results in minor malaria transmission until June [18]. Major factors that contributed to malaria endemicity in the areas are elevation from 1436 to 1850 meters above sea level, seasonality of rainfall patterns, average annual temperature that ranges from 16 to 32°C, and various microecological factors that favor mosquito breeding [18–20].

### 2.2. Sample Collection and Processing

A total of 148 blood spotted samples (DBS) were collected from September 2019 to August 2020, from *P. falciparum*-infected malaria patient in the study area. The age of study participants ranged from one to sixty-six presenting to selected health facilities. The inclusion criteria for this study were uncomplicated malaria cases due to *P. falciparum* and having history of fever onset since 24 hours of the clinical examination. Health facilities were selected based on a record of higher patient caseloads at their respective location. Through finger prick, *P. falciparum*-positive blood samples were spotted on Whatman™ 3MM filter paper for DBS preparation, by trained laboratory technologists, and it is temporarily stored together with relevant patient data. The prepared DBS is later transported to the Adama regional laboratory research center and kept at -20°C prior to molecular laboratory work. Malaria microscopy was conducted for symptomatic patients as per the national parasitological diagnosis of malaria at each health facility. The identified falciparum-positive microscopic slides were collected from sample collection sites for further analysis. Two independent WHO-certified laboratory technologists for malaria microscopy proofread the slides for malaria parasite species identification and parasitaemia level, at the Adama malaria diagnostic center. Parasite density was calculated by averaging the two closest counts. The parasite density per microliter (*μ*l) of blood was estimated by counting the number of WBC by the field examined assuming that 8000 WBC were present in 1 *μ*l of blood. The number of parasite density per microliter (*μ*l) of blood was calculated by using the following formula [21, 22]:

(1) $$\begin{array}{c} \text{Parasite density}/\mu \text{l}=\frac{\text{Number of parasite counted per }200\text{ WBC}\times \text{assumed WBC}/\mu \text{l}}{\text{Number of WBC counted}}. \end{array}$$

For comparison with ranked-order variables during data analysis, parasitaemia were categorized into five levels: L1 (<50 parasites/*μ*l blood), L2 (50-499 parasites/*μ*l blood), L3 (500-4999 parasites/*μ*l blood), L4 (5000-49999 parasites/*μ*l blood), and L5 (≥50,000 parasites/*μ*l blood) [23].

### 2.3. Extraction of *P. falciparum* DNA and Amplification of the *msp-2* Gene

The parasite genomic DNA extraction and its *msp-2* gene amplification were conducted at malaria research laboratory, Aklilu Lemma Institute of Pathobiology, Addis Ababa University (AAU). The extraction of genomic DNA of *P. falciparum* DBS was carried out by using 0.5% Tween® 20 (Sigma-Aldrich, USA) to lyse RBC and tracked by treatment with 6% Chelex® 100 (Sigma-Aldrich, USA) in a water bath at 96°C [14].

The polymorphic region of the *P. falciparum msp-2* gene (block 3) was used as a genetic marker for the genotyping of parasite populations. Nested PCR of the *msp-2* (block 3) polymorphic region was performed by slight modification of primers and methods from the previously described reports [14, 24] (Supplementary 1). In brief, initial amplification (N1) of the msp-2 gene was carried out in a final volume of 20 *μ*l amplification mixture containing 1x FIREPol® Master Mix (4 *μ*l), 0.5 *μ*l of each primer (M2-OF and M2-OR), 11 *μ*l of nuclease-free water aliquot, and 4 *μ*l of DNA template. In the second (N2) amplification reaction, 20 *μ*l amplification mixture containing 1x FIREPol® Master Mix (4 *μ*l), 0.5 *μ*l of each primer FC27 (B1)/3D7 (A1) and FC27 (B2)/3D7 (A2), and 13 *μ*l of nuclease-free water aliquot to 18 *μ*l to which 2 *μ*l of DNA template was added for the amplification of each allelic variants was used.

The PCR amplification profile for both N1 and N2 reactions includes initial denaturation at 95°C for 3 minutes, denaturation at 94°C for 1 minute, annealing at 58°C for 1 minute, elongation at 72°C for 2 minutes, and final elongation for 5minutes. Allele-specific positive control 3D7 and DNA-free negative control were used in each set of the reactions. Gel-electrophoresis of the DNA fragment for *msp-2* allelic families was performed on 2% agarose gel stained with ethidium bromide and visualized under a Benchtop 2UV trans-illuminator (UVP) (USA) and photographed to estimate band size in relation to the 50 bp DNA ladder (Invitrogen, by Thermal Fisher Scientific) (Supplementary 2). The detection of one PCR fragment on each locus indicates that infection is monoclonal, whereas the presence of more than one fragment on each locus shows polyclonal infection [25]. When the size of the allele fragment was found to be less than 20 bp interval, they were considered the same [26].

### 2.4. Data Analysis

Multiplicity of infection (MOI) is defined as the number of distinct parasite genotypes coexisting within the given infection. In the present study, it is based on genotyping of the *msp-2* block 3 gene. Thus, MOI for *msp-2* for each clinical isolate represents the number of PCR fragments obtained from 3D7 and FC27 gels [27]. Each round of PCR amplification targeting the allelic variant form using specific primers reveals several variant forms that differ in fragment size due to the variation in repeat allotypes encoding a single amino acid motif that characterizes the variant family [20, 27]. Amplification and gel-electrophoresis of *msp-2* alleles in the study revealed 113 and 102 fragments for 3D7 and FC27 genotypes, respectively. Thus, mean MOI could be estimated by dividing the total number of gel fragments detected in the *msp-2* gene by the total number of positive samples in the same marker [28, 29], which indicates the average number of genotypes per infected subject.

Allele frequency represents the incidence of the allelic variant (3D7 or FC27) in the sample isolates. It could be determined by dividing the number of times the allele of interest (3D7 or FC 27) is observed in a population by the total number of copies of all alleles detected for the *msp-2* gene in the isolates. Descriptive statistics were used to calculate the frequency of each *msp-2* allelic family in relation to the total number of genes successfully amplified for that locus. Size polymorphism in each allelic family shows that one band represents one amplified PCR fragment derived from a single copy of the *P. falciparum msp-2* gene. When alleles in each family were less than 20 bp, they were considered the same. The Pearson chi-square test was conducted for statistical comparison of categorical variables. *P* < 0.05 was used to test the level of statistical significance to accept or reject the hypothesis. All statistical tests were performed by using SPSS version 20.0 (SPSS Inc., Chicago, USA). In addition, geometric mean of parasite density was calculated by using the Microsoft Excel 2016 version.

The heterogeneity index (He) representing the probability of being infected by two different populations of *P. falciparum* with different alleles at a given locus was calculated by the formula:

(2) $$\begin{array}{c} \text{He}=\left(\frac{n}{n-1}\right)\left(1-\sum p^{2}\right), \end{array}$$

where *n* is the number of the isolates analyzed and *p* represents the frequency of each different allele at a locus.

## 3. Results

### 3.1. Sociodemographic and Parasitological Data of Study Participants

148 samples of *P. falciparum* clinical isolates were successfully amplified for the *msp-2* gene. Of which, 105 (71%) were males. The age of the study participant ranged from 1 to 66 (mean ± SD) (27.0 ± 13.6^∗^) years. Parasite density ranged from 64 to 104,320 parasites/*μ*l with a geometric mean of 5654 parasites/*μ*l. Of all study participants, 85 (57%) were from urban inhabitants. Of all the study subjects by occupation, students, daily laborers, and farmers alone accounted for 113 (76%) *P. falciparum* malaria cases in the study area. Moreover, *P. falciparum* infection was found to be significantly related (*X*^2^ = 0.017) to occupation type of the patients (Table 1).

From the total successfully amplified *msp-2* gene, 85 (57.4%) isolates were from urban inhabitants and 57 (38.5%) of the isolates from Adama (Table 2). Patient data was recorded as urban or rural inhabitants based on the administrative structure of the locality [30]. The spatial feature of the distribution of the *P. falciparum msp-2* gene allelic variant across sample collection sites indicated variation (Table 2), which was statistically significant (*X*^2^ (8) = 29.6, *P* ≤ 0.001). This shows heterogeneity in its distribution.

### 3.2. Allele Frequency, Genetic Diversity, and Multiplicity of Infection

Allele genotyping of the *msp-2* gene of *P. falciparum* in Adama and its surroundings revealed its polymorphic nature. The frequency of 3D7 and FC27 was 31.8% (47/148) and 27.7% (41/148), respectively. Moreover, 40.5% (60/148) carried both 3D7 and FC27 alleles, with mean MOI of 1.4 and the heterogeneity index (He) of 0.49 (Table 3). In addition, size polymorphism demonstrated in the study revealed that 106 and 99 samples were successfully genotyped for 3D7 and FC27 variant forms, respectively. In this study, among the *msp-2* isolates, seven 3D7 (200-700 bp) and ten FC27 (250-700 bp) different size polymorphic alleles were observed.

Comparison of geometric mean of the parasite density at different age groups showed disproportionate burden of parasitaemia in school-age children. The overall frequency of the *msp-2* gene allelic variant among symptomatic patients tends to increase with age groups, although the variation was not statistically significant (*X*^2^ = 0.09). Likewise, though not linearly, the mean MOI slightly increased with age groups (Table 4).

**Figure 2:**
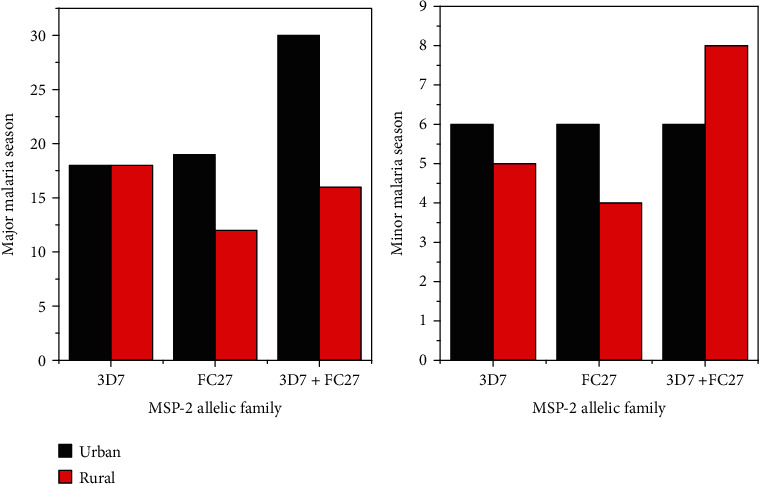
Urban-rural and seasonal features of the *msp-2* allelic variant of *P. falciparum* isolates from September 2019 to August 2020, in the study area (*n* = 148). Analysis of the *P. falciparum msp-2* gene allelic variant in relation to parasitaemia level for patients enrolled in the study has shown an overall increment with clinical prevalence of the parasitaemia level from 500 to 49,000/*μ*l of blood (Figure 3).

Moreover, analysis of the urban rural distribution of the *msp-2* gene allelic variant during the major and minor malaria season in the study area nearly depicts analogous transmission patterns (Figure 2), showing no statistically significant variation (*X*^2^ (2) = 1.1, *P* = 0.56).

## 4. Discussion

Ethiopia has now moved forward in targeting a nationwide malaria elimination program by 2030. For effective implementation of this strategic target, one of the key intervention strategies is improving malaria surveillance and response [31]. In this regard, a molecular epidemiological study approach like characterization of the block 3 region of *P. falciparum msp-2* provides comprehensive molecular evidence for effective disease surveillance that could be ultimately translated to core interventions in the control and eventual elimination of malaria.

The present study revealed consistence with our previous report [32] and that of Golassa and White [18], where the incidence of *P. falciparum* isolates in the study area was higher in male (71%) (Table 1). The major factors that may account for such higher malaria cases compared to female are due to special risk of male individual to malaria infection from occupational and travel-related factors [31] in Ethiopian tradition. The incidence of *P. falciparum* isolates in the present study was significantly related to occupation type (*X*^2^ = 0.017) (Table 1) which is in agreement with the review report of Degarege and his colleagues [33]. This could be due to strong relation of malaria incidence with lower standard of living that contributed to the occurrence of 76% of all *P. falciparum* isolates in the present study from farmers, daily laborers, and students alone (Table 1).

Investigation of the *msp-2* block 3 region of the *P. falciparum* genetic profile revealed in this study was the first in its kind in the study area. The authors examined seasonal and spatial distribution of *msp-2* allelic variants in the study area (Figure 2), together with their urban-rural counterparts (Table 2). Of the total successfully genotyped *msp-2* gene, the monoclonal alleles of 3D7 and FC27 constitute 31.8% and 27.7%, respectively (Table 3). Report from the maritime region of Togo [34] and Ponte-Noire, Republic of Congo [35], complements this finding. Moreover, the number of *msp-2* genotypes detected for 3D7 and FC27 was 7 and 10, respectively, although the number of genotypes might have been underestimated due to the limitation of the techniques. Fragment size polymorphism described in the current study is nearly comparable with the previous report from Republic of Congo [35], Nigeria [36], Sudan [21], and northeastern Ethiopia [28]. Other reports from Brazzaville, Congo [8], and northwestern Ethiopia [37] showed the predominance of the 3D7 allelic family. Such inconsistency in *P. falciparum* allelic size polymorphism could be due to local malaria epidemiology and scope of sample population covered in the study. The rate of *msp-2* multiple genotype infection was 40.5% (Table 4), which reflects the transmission status of the parasite population in the study area. The overall mean MOI was 1.4. This finding is lower than the previous report from southwestern Ethiopia [29], northwestern Ethiopia [37], Sudan [21], Cameroon [38], and Nigeria [39], but somewhat higher than the previous report from northeastern Ethiopia [28] and Ghana [40]. The variation in multiclonal infection and mean multiplicity of infection could be due to the level of malaria endemicity, local transmission settings, and the age structure of the population [27, 41]. The overall mean MOI identified in the present study could serve as proxy of transmission intensity for targeted intervention in the region. Furthermore, the present study showed the heterogeneity index (He = 0.49), implicating nearly intermediating genotype diversity within the *msp-2* locus in the study area, compared to the previous report from southwestern Ethiopia [42], northwestern Ethiopia [37], Cameroon [38] and Malaysia [43].

**Figure 3:**
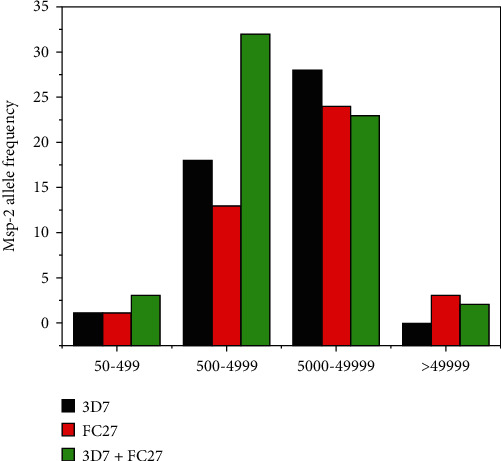
Comparison of the *msp-2* gene allelic variant from malaria patients due to *P. falciparum*, who attended to different health facilities in the study area from September 2019 to August 2020 (*n* = 148).

In the current study, analysis of the *msp-2* gene allelic variant in the urban and its surrounding rural area (Figure 2) during the major and minor malaria transmission seasons [44] revealed an analogous transmission pattern, having no statistically significant variation (*P* = 0.54). In complement with this finding, Funwei and his colleagues [36] reported similar level of genetic diversity in urban and rural areas. On the other hand, Soulama and his colleagues [41] reported that the *msp-2* allelic variant and its multiple infections vary from urban-rural location depending on transmission intensity and independent of seasonal change. The major factor that might have contributed to such inconsistent report could be differences in local epidemiology of malaria parasite flow between urban and its surrounding rural areas. In the present study, analysis of the variation of *msp-2* allelic frequency across different age groups has shown increment in parallel with *P. falciparum* clinical prevalence during the study period. Moreover, in this study, mean MOI slightly increased with age when compared with younger children (Table 4). This finding differs from the previous report from the hyperendemic area of Burkina Faso [20], where negative relationship between mean MOI and patient age was reported. However, consistent with the present finding, report from Tanzania [45] and Burkina Faso [41] revealed that, as immunity develops, mean MOI seems to increase contributing to the rise in mean MOI. Thus, the relationship between the levels of immunity and MOI needs further investigation. Even though different factors may contribute to the fluctuation of parasitaemia level in symptomatic patients over time, the highest geometric mean of microscopically detected parasitaemia level in school-age children revealed in this study (Table 4) might be due to delayed acquisition of protective immunity of this age group [46]. Moreover, consistent with the report from Equatorial Guinea [24], the present study revealed the existence of direct relation with *msp-2* allele frequency and multiple infections with the clinical prevalence of the parasitaemia level (Figure 3 and Table 4). This could be due to the level of acquired immunity in malaria endemic setting of the study area. Furthermore, district-wise distribution of the *msp-2* allelic variant and multiple infections in the study area (Table 2) showed a highly significant variation (*P* ≤ 0.001). This could be due to differences in local microecological factors, change in parasite vector interaction, and spatial heterogeneity of the study sites under consideration [47, 48].

Even though, this is the first study to analyze *P. falciparum* genetic diversity in Central Ethiopia by using one of the most polymorphic msp-2 genes, the study has some technical limitations. Firstly, the association between the observed dominant allelic families and disease severity was not examined, because all samples were collected from uncomplicated malaria patients. Secondly, the number of alleles maybe underestimated due to the limitations of techniques (nested PCR amplification and gel-electrophoresis) used. Therefore, further characterization of such polymorphic region needs to be designed by increasing the sample size and implementation of advanced techniques like analysis of microsatellite markers followed by capillary electrophoresis and DNA sequencing analysis for samples isolated from symptomatic and asymptomatic cases for further study.

## 5. Conclusions

The present study revealed a nearly moderate transmission rate of *P. falciparum* clinical isolates among symptomatic malaria patients. Therefore, to sustain such declining phase of malaria transmission in Central Ethiopia, in addition to the classical epidemiological study approach, the ongoing malaria control and elimination program should be accompanied by similar molecular surveillance for targeted intervention. Moreover, the present study revealed the absence of significant variation in allele frequency in both urban and its rural counterpart and similar allele frequency during the major and minor malaria season, reflecting frequent inbreeding among the existing parasite strains in both settings. This indicates strong evidence for the presence of similar malaria epidemiology in the urban and rural location of the study area demanding a similar intervention strategy. Thus, the findings described in this study will serve as baseline molecular evidence for further research on areas having similar malaria epidemiology to make the control and elimination efforts effective.

## Figures and Tables

**Table 1:** Sociodemographic characteristics and parasitological data of symptomatic malaria patients due to *P. falciparum* clinical isolates genotyped for the *msp-2* gene from Adama and its surroundings (*n* = 148).

Patient characteristics	Sample genotyped	Chi-square (*X*^2^^)^
Mean age (year)	27.0 ± 13.6^∗^ (SD)	
Age range (year)	1-66	0.09
Sex ratio (male/female)	105/43	0.18
Residence (urban/rural)	85/63	0.56
Occupation		
Farmer	31 (21%)	0.017
Housewife	12(8%)	
Daily laborer	35 (23.8%)	
Government employee	13 (8.8%)	
NGO employee	2 (1.3%)	
Business man	7 (4.7%)	
Student	47 (32%)	
Geometric mean of parasitic density (P/*μ*l) of blood	5,654	
Parasite density range (P/*μ*l) of blood	64–104,320	
Parasitaemia level		
(i) 50-499 P/ul of blood	5 (3.3%)	0.075
(ii) 500-4999 P/ul of blood	63 (42%)	
(iii) 5000-49,999 P/ul of blood	75 (50.6%)	
(iv) 50,000 P/ul of blood	5 (3.3%)	

**Table 2:** Urban and rural distribution of *P. falciparum msp-2* alleles isolated from symptomatic patients across sample collection sites from Adama and its surroundings (*n* = 148).

msp-2 alleles	Adama		Modjo		Wonji		Melkasa		Olanciti	
	Urban	Rural	Urban	Rural	Urban	Rural	Urban	Rural	Urban	Rural
3D7	17	6	0	0	1	4	0	5	6	8
FC27	17	2	4	2	2	3	0	3	2	6
3D7+FC27	13	2	10	5	2	1	5	13	6	3
Total	47	10	14	5	5	8	5	21	14	17

**Table 3:** Genetic diversity, allelic frequency, and fragment size data of the block 3 region of the *msp-2* gene isolated from symptomatic malaria patients due to *P. falciparum*, from Adama and its surroundings, Oromia, Ethiopia (*n* = 148).

Msp-2 alleles (*n* = 148)	Allele frequency *n* (%)	Fragment size (bp)	Number of alleles	Mean MOI	Heterogeneity index (He)
3D7	47 (31.8)	200-700	7	1.4	0.49
FC27	41 (27.7)	250-700	10		
3D7+FC27	60 (40.5)				
Total	148				

**Table 4:** Distribution of *msp-2* allelic variants, mean MOI, and geometric mean of parasite density among different age groups of malaria patients due to *P. falciparum* in Adama and its surroundings (*n* = 148).

Characteristics	Age groups in year				
	<5, *n* (%)	5-14, *n* (%)	15-24, *n* (%)	>24, *n* (%)	Total *n*
3D7	2 (4.2)	4 (8.5)	10 (21)	31 (66)	47
FC27	2 (4.8)	7 (17)	14 (34)	18 (44)	41
3D7+FC27	1 (1.7)	6 (10)	28 (47)	25 (41.7)	60
Total	5 (3.4)	17 (11)	52 (35)	74 (50)	148
Mean MOI	1.2	1.5	1.5	1.4	
Geometric mean of parasite density	6455	7419	4324	6381	

## Data Availability

Data used to support this study are fully available within the manuscript and detailed data are available from the corresponding author upon request.

## Supplementary Materials

Supplementary 1 Primer design.

Supplementary 2
*msp-2* allelic fragment size using a 50 bp ladder identified by gel electrophoresis.
