# Supplementary material for: Plasmodium falciparum Clinical Isolates Reveal Analogous Circulation of 3D7 and FC27 Allelic Variants and Multiplicity of Infection in Urban and Rural Settings: The Case of Adama and Its Surroundings, Oromia, Ethiopia
Source: J Parasitol Res. 2022 Mar 14;2022:5773593. doi: 10.1155/2022/5773593 (PMC8966748; doi:10.1155/2022/5773593)
Supplement: Supplementary 1 — Primer design. [file 5773593.f1.docx]

| S.N | Primer name | Sequence | Target gene/allele |
| --- | --- | --- | --- |
| Amplification of msp-2 gene | M2 - OF | 5’-ATG AAG GTA ATT AAA ACA TTG TC TA-3’ | MSP-2 |
|  | M2-OR | 5’- CTT TGT TAC CAT CGG TACA TT CTT-3’ |  |
| Msp- 2 allelic families | FC27 (B1) | 5’-GCA AATGAA GGT TCT AAT ACT AAT AG-3’ | FC27 allele |
|  | FC27 (B2) | 5’- GCTTTGGGTCCTTCTTCAGTTGATTC-3’ |  |
|  | IC3D7 (A1) | 5’-GCA GAA AGT AAG CCT TCT ACT GGT GCT-3’ | IC3D7 allele |
|  | IC3D7 (A2) | 5’- GATTTGTTTCGGCATTATTATGA-3’ |  |
