# Supplementary figures and images for: Plasmodium falciparum Clinical Isolates Reveal Analogous Circulation of 3D7 and FC27 Allelic Variants and Multiplicity of Infection in Urban and Rural Settings: The Case of Adama and Its Surroundings, Oromia, Ethiopia
Source: J Parasitol Res. 2022 Mar 14;2022:5773593. doi: 10.1155/2022/5773593 (PMC8966748; doi:10.1155/2022/5773593)

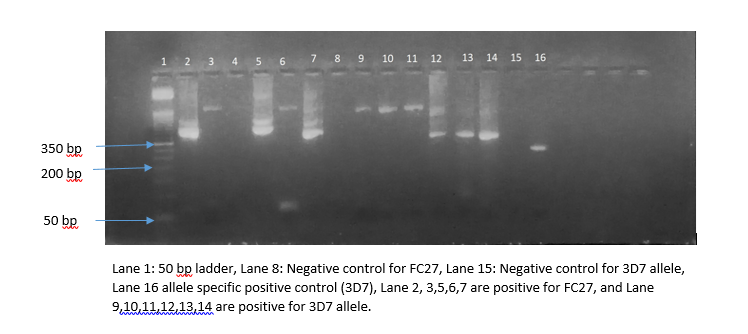

Supplement: Supplementary 2 — msp-2 allelic fragment size using a 50 bp ladder identified by gel electrophoresis. [file 5773593.f2.docx]
